# Post-COVID-19 Granulomatous Inflammation in the Lung, Distinct From Sarcoidosis: A Report of Two Cases

**DOI:** 10.7759/cureus.50821

**Published:** 2023-12-20

**Authors:** Keisuke Watanabe, Okudela Koji, Mai Matsumura, Takeshi Kaneko

**Affiliations:** 1 Department of Pulmonology, Yokohama City University Graduate School of Medicine, Yokohama, JPN; 2 Department of Pathology, Yokohama City University Graduate School of Medicine, Yokohama, JPN

**Keywords:** lymphoplasmacytic infiltration, non-caseous granuloma, fibroblastic proliferation, coronavirus disease 2019, post-covid-19, granuloma, autoimmune disease, sarcoidosis, covid-19

## Abstract

This report describes two cases of granulomatous lung inflammation following coronavirus disease 2019 (COVID-19), presenting as a sarcoidosis-like reaction with granuloma formation in airspaces and interstitium. Clinical and pathological findings in both cases were similar to but still distinct from sarcoidosis. In the first case, the chest CT of a 55-year-old male with a history of polymerase chain reaction (PCR)-confirmed COVID-19 showed well-defined multiple nodules in the bilateral lung fields. He underwent video-assisted thoracic surgery for diagnostic purposes. The pathological specimen showed loose non-caseous granulomas with mild lymphoplasmacytic infiltration and early fibroblastic proliferation in alveolar spaces. In the second case, a 68-year-old male, who presented with consolidation in the anterior segment of the right upper lobe, underwent bronchoscopy and transbronchial lung biopsy showed non-caseous granulomas with mild lymphoplasmacytic infiltration in the peribronchiolar interstitium. The opacities improved spontaneously in both cases. Further studies are needed to determine whether COVID-19 could cause granulomatous lung inflammation distinct from sarcoidosis.

## Introduction

The coronavirus disease 2019 (COVID-19) pandemic is settling with the development of vaccines and therapeutic medications. However, long COVID, an umbrella term for symptoms and conditions that develop after acute COVID-19, still affects many worldwide. In addition to the long COVID possibility, COVID-19 increases the risk of autoimmune diseases [1-3]. A recent study reported an increased risk of sarcoidosis following COVID-19 [3].

Here, we report two cases of granulomatous lung inflammation following COVID-19, with a sarcoidosis-like reaction presenting with granuloma formation in airspaces and interstitium. In these cases, clinical and pathologic findings were similar to but distinct from sarcoidosis.

## Case presentation

Case 1

A 55-year-old male presented with a headache, limb pain, and cough in March 2023. He had the diagnosis of polymerase chain reaction (PCR)-confirmed COVID-19 in December 2022. He recovered without antiviral medication or systemic steroids. His medical history is unremarkable except for hypertension. He smoked 20 cigarettes per day from age 19 to 55. He worked as a taxi driver and had no record of dust exposure. In light of this, long COVID was suspected.

**Figure 1:**
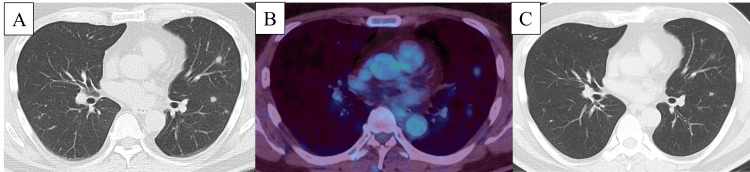
Radiological findings in Case 1. Chest CT showed multiple well-defined nodules in bilateral lung fields (A) and PET-CT showed fluorodeoxyglucose uptake in multiple lung nodules (B) and left hilum lymph nodes. Multiple nodules improved spontaneously (C). PET: positron emission tomography

Physical examination revealed no rales or palpable lymph nodes. Chest CT showed bilateral well-defined nodules in lung fields (Figure 1A). Serum beta-D-glucan, *Aspergillus* antigen, *Cryptococcus* antigen, blood T-cell spot test for tuberculosis, and anti-GPL-core IgA antibody were negative. Angiotensin-converting enzyme (ACE) and anti-neutrophil cytoplasmic antibody did not increase. Serum cytomegalovirus antigen and human T-cell leukemia virus type 1 antibody were also negative. positron emission tomography (PET)-CT showed fluorodeoxyglucose (FDG) uptake in multiple lung nodules and left hilum lymph nodes (Figure 1B).

**Figure 2:**
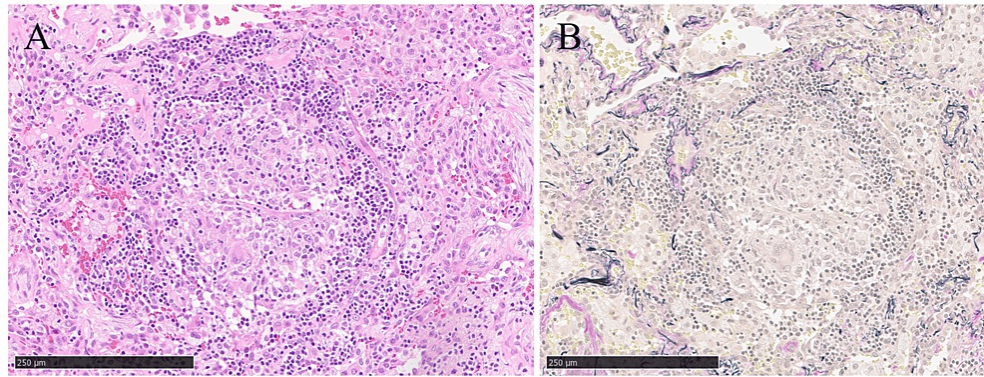
Pathological findings in Case 1. Loose granulomatous assembly of histiocytes with mild lymphoplasmacytic infiltration and young fibroblastic proliferation is found in alveolar spaces (A, hematoxylin and eosin; B, Elastica van Gieson).

The patient underwent diagnostic video-assisted thoracic surgery in May 2023. No bacteria or fungi were detected with tissue culture. The pathology specimen showed loose non-caseous granulomas with mild lymphoplasmacytic infiltration and early fibroblastic proliferation in alveolar spaces (Figure 2). Multiple nodules improved spontaneously within six months (Figure 1C).

Case 2

A 68-year-old male presented with consolidation in his right lung in June 2021. His opacity was detected incidentally with follow-up of COVID-19 and he had no symptoms. His medical history was unremarkable except for allergic rhinitis. The patient was an office worker, had no dust exposure, and smoked 30 cigarettes per day for 40 years until the age of 63. He had been diagnosed with PCR-confirmed COVID-19 in March 2021. His condition recovered without systemic steroids or antiviral medication.

**Figure 3:**
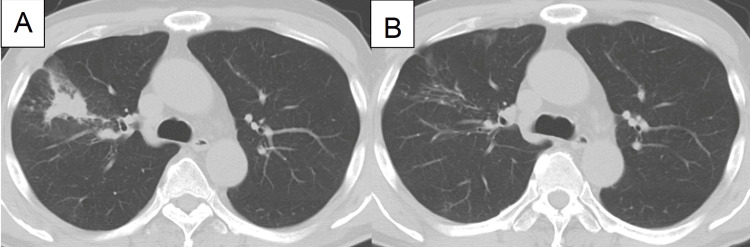
Radiological findings in Case 2. Chest CT showed consolidation in  the anterior segment of the right upper lobe (S3) (D) and the opacity was improved spontaneously (E).

Physical examination did not reveal any rales or palpable lymph nodes. The chest radiograph showed consolidation between his upper and middle fields. Chest CT showed consolidation in the anterior segment of the right upper lobe (Figure 3A).

**Figure 4:**
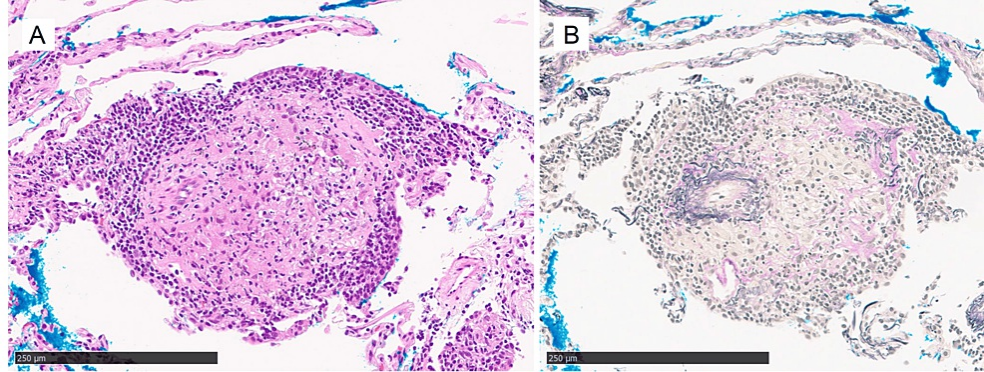
Pathological findings in Case 2. Non-caseous granuloma with mild lymphoplasmacytic infiltration is seen in peribronchiolar interstitium (C, hematoxylin and eosin; D, Elastica van Gieson). The scale bars show 250µm.

Serum ACE and soluble IL-2 receptor levels were not increased. Blood screening for fungi, including beta-D-glucan, *Aspergillus* antigen, *Aspergillus* antibody, and *Cryptococcus* antigen was negative. The patient underwent bronchoscopy and a transbronchial lung biopsy was performed, showing non-caseous granulomas with mild lymphoplasmacytic infiltration in the peribronchiolar interstitium (Figure 4). The opacity improved spontaneously over four months (Figure 3B), and he had no recurrence during a follow-up of two years.

## Discussion

Here, we reported two cases of granulomatous lung inflammation following COVID-19. A recent study reported an increased risk of sarcoidosis following COVID-19. Additionally, there have been several case reports of pulmonary sarcoidosis occurring several weeks to months after COVID-19 [4-8].

In Case 1 of the current report, granulomas in airspaces were not typical for sarcoidosis, and an airway stimulation pathogenesis like hypersensitivity pneumonia was suggested. ACE level was not elevated and extrapulmonary lesions were not detected in either of these two cases. Therefore, it is uncertain whether these cases constitute sarcoidosis or another sarcoidosis-like syndrome. This recalls a similar dilemma in pediatric patients between multisystem inflammatory syndrome in children (MIS-C) and Kawasaki disease. After COVID-19, MIS-C, which resembles Kawasaki disease, was reported [9,10]. There are some similarities between MIS-C and Kawasaki disease, but MIS-C was recognized as an entity different from Kawasaki disease [10]. Similarly, COVID-19 could cause granulomatous lung inflammation distinct from sarcoidosis. Further studies are needed to clarify the nature of pulmonary granulomatous lesions after COVID-19.

Clinical features of granulomatous lung inflammation post COVID-19 must be clarified. Radiological findings differ between these two cases and thus such post-COVID-19 granulomatous lung inflammation could show a variety of radiological findings. In addition, the natural course of granulomatous lung inflammation after COVID-19 is also uncertain. Spontaneous resolution in Cases 1 and 2 indicates that the course is self-limiting; thus, many cases might have been overlooked.

## Conclusions

We reported two cases of granulomatous lung inflammation following COVID-19. This could be a possible new post-COVID-19 disease entity. Further studies are needed to know whether COVID-19 could cause granulomatous lung inflammation distinct from sarcoidosis.
